# Experimental Characterization of Single-Color Power LEDs Used as Photodetectors

**DOI:** 10.3390/s20185200

**Published:** 2020-09-11

**Authors:** Jan Sticklus, Peter Adam Hoeher, Martin Hieronymi

**Affiliations:** 1 GEOMAR Helmholtz Centre for Ocean Research Kiel, 24148 Kiel, Germany; 2 Faculty of Engineering, University of Kiel, 24143 Kiel, Germany; ph@tf.uni-kiel.de; 3 Institute of Coastal Research, Helmholtz-Zentrum Geesthacht, 21502 Geesthacht, Germany; martin.hieronymi@hzg.de

**Keywords:** LED used as photodetector, visible light communication, optical bandpass filtering

## Abstract

Semiconductor-based light emitting diodes can be used for photon emission as well as for detection of photons. In this paper, we present a fair comparison between off-the-shelf power Light emitting diodes (LEDs) and a silicon photodetector with respect to their spectral, temporal, and spatial properties. The examined LED series features unexpected good sensitivity and distinct optical bandpass characteristic suitable for daylight filtering or color selectivity. Primary application is short range optical underwater communication, but results are generally applicable.

## 1. Introduction

Light emitting diodes (LEDs) are designed as light sources—for instance, see [1]. Advantages of group III–V compound semiconductor LEDs compared to incandescent/halogen/fluorescent illuminants include: LEDs exhibit a higher luminous efficacy (in lm/W); they are more reliable in terms of lifetime; the switching speed is much faster; the form-factor is design-friendly; LEDs are available in many different colors; and they operate at lower voltage.

However, III–V LEDs can also be used as photodetectors (PD), although they are not optimized for this purpose. This dual-use of solid-state light emission and detection has been published in the 1970s by Forrest W. Mims [2,3] but was rarely put into practice for the next 30 years, with a few exceptions. The responsivity (in A/W) of LEDs is wavelength-dependent. As photodiodes, LEDs are sensitive to wavelengths equal to or shorter than the peak wavelength they emit, [4]. Green LEDs are sensitive to blue and partially green light, but not to yellow and red light. Mims et al. used this characteristic to construct a sunlight photometer [3]. The breakthrough came in 2003, when Dietz et al. proposed to use LEDs as bidirectional transceivers for low-cost digital communication applications [5], nowadays known as LED-to-LED communication. Subsequently, a large variety of sensor applications have been investigated, where LEDs are employed as photodetectors. For example, LED arrays can be employed as touch-sensitive input and output devices that register reflected light from a finger or a stylus as invented by Hudson [6]. Shepherd et al. applied LEDs as low-cost surface-mount gas sensors [7]. Ben-Ezra et al. used the spectral response to design a bidirectional reflectance distribution function measurement device consisting exclusively of LEDs [8]. Macka et al. proposed LEDs for analytical chemistry [9]. Besides these numerous sensor applications, LED-to-LED communication is still under investigation. The diversity of consumer electronic applications is huge, ranging from toy-to-toy communication employing low data rates [10] to high-speed applications with data rates beyond 100 megabits per second (Mbps) [11,12]. In order to overcome the bandwidth limitation of LEDs, multicarrier modulation in [11] and receiver-side digital equalization in [12] are applied. A gigabit per second (Gbps) visible light communication (VLC) system based on yellow LEDs as receiver is introduced in [13], and, in [14,15], a communication system by which two LED devices are used for two-way visible light communication while also providing illumination. Visible light communication and related tasks—like spectral-based intensity detection for the purpose of high-quality human central lighting—are among the driving forces in LED research [16].

Because LEDs are not intended to be used as photodetectors, manufacturers do not provide specifications about their response function, spectral sensitivity, or dynamic range [8]. For this reason, an experimental characterization of LEDs is necessary when used as photodetectors [17].

Original contributions of this article include:

- The wavelength-dependent responsivity (in A/W) is determined for single-color power LEDs when used as photodetectors. Power LEDs are shown to behave quite differently compared to low-power LEDs probed so far.
- The spectral sensitivity is compared with a typical silicon PD and the theoretical bound. It is shown that the gap with respect to the optimum responsivity is small.
- The dynamic behavior in terms of rise/fall time and junction capacitance is investigated.
- The optical field of view (FOV) is compared for emitter and detector mode.
- The impact of light polarization is tested.

Experimental results are obtained for two off-the-shelf color LED series with flat lensless surfaces. Unlike low-power LEDs studied in previous publications (see e.g., [17]), no plastic lenses needed to be rubbed off and the photosensitive area (necessary for a computation of the responsivity) could be precisely taken from the datasheet. Our main emphasis is on optical underwater applications [18,19] because, in this area, single-color high power LEDs are needed and because optical (colored glass or thin film) filtering for the purpose of ambient light suppression is troublesome. However, the results reported next are universally applicable.

The remainder is organized as follows. In Section 2, the experimental setups under investigation are presented. Numerical results are reported in Section 3. Finally, conclusions are drawn in Section 4.

## 2. Description of Experimental Setups

**Figure 1:**
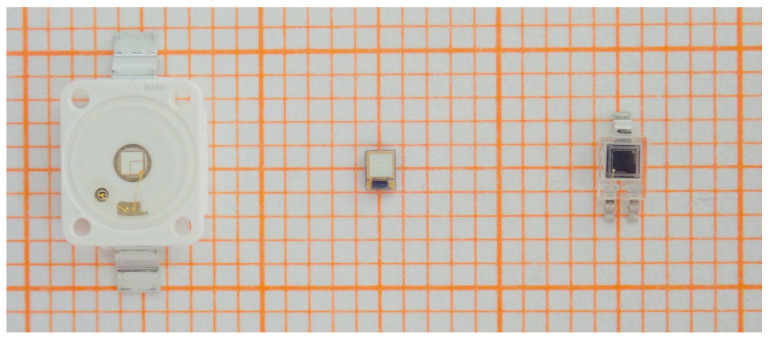
This picture shows an LED of the Osram Golden Dragon series on the left side, an LED of the Lumileds Z series in the middle, and the reference photodetector Osram SFH 2400 on the right side. The grid size is 1 by 1 mm.

For visible light communications, particularly in underwater applications, single-color power LEDs are the first choice for low-cost and short-range applications. Compared to white LEDs, the market only offers a limited selection of single-colored power and high-power LEDs, respectively. Regarding the suitability as photodetector, multiple-die and converted types are beyond the scope of this contribution. Popular low-cost single-die power LEDs in the 1 mm${}^{2}$ chip size class include the Osram Golden Dragon series (Osram Semiconductor, Regensburg, Germany) and the Lumileds Luxeon Z color series (Lumileds Holding BV, Schipohl, The Netherlands), see also Figure 1, the latter offered in a rarely found large variety of colors. The few high-power LEDs on the market with larger single dies, for example a 12 mm${}^{2}$ series offered by Luminus (Luminus Inc., Sunnyvale, CA, USA), are mostly available only in red, green, and blue colors. This generally high-priced segment is not necessarily interesting for a second application as PD. The two series mentioned, Osram Golden Dragon [20] and Lumileds Luxeon Z [21], and the silicon positive intrinsic negative (Si PIN) photodetector Osram SFH 2400 [22] were chosen for direct comparison, and they are specified in Table 1 and Table 2. They have the same active area and shape and are all planar types without primary optics, offering a typical FOV of 120° full width half mean (FWHM). This selection therefore allows comparative measurements to be carried out under repeatable conditions. Experiments were conducted without applying a reverse voltage to the device under test (DUT).

### 2.1. Spectral Measurements

**Figure 2:**
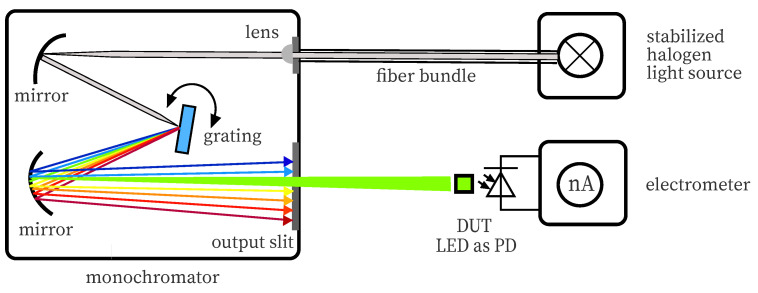
This figure illustrates the measurement setup to determine the spectral sensitivity of a photodetector or an LED used as photodetector. The light source is connected via a fiber bundle to the monochromator. The light output of the monochromators adjustable spectrum hits the device under test. The generated photocurrent is quantified by a nanoampere meter.

To determine the spectral sensitivity of a photodetector, monochromatic light or light with a small optical bandwidth is needed. In order to maintain this, a light source with a wide spectrum like a halogen bulb can be combined with a monochromator that is separating the wavelength of the light spatially. For our experiments, a grating type monochromator Oriel 77250 (Newport Corporation, Irvine, CA, USA) was used in combination with halogen source Schott KL1500 (Schott AG, Mainz, Germany), see also Figure 2. The externally stabilized light source was operated at 70 W with a usable continuous spectrum of approximately 400 nm to 750 nm. The light of the monochromators’ output hitting the 1 by 1 mm active area of the DUT at a distance of 50 mm has an optical bandwidth of a few nanometers. The generated photocurrent is measured directly by a Keithley 6517 electrometer (Keithley, Solon, OH, USA) in the nanoampere range at a monochromator step size of 10 nm.

### 2.2. Temporal Measurements

**Figure 3:**
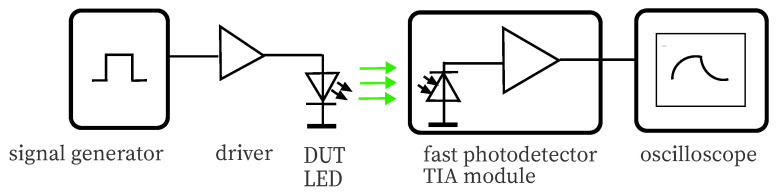
This drawing shows the setup for measuring the rise/fall time of LEDs used as light source. The source part of this setup has also been used for investigation of the temporal characteristics of the LEDs used as photodetector.

The bandwidth of a photodetector can be determined by measuring the rise time $t_{rm}$ of the impulse response. Ideally, a fast light source providing a rise time $t_{rs}\ll t_{rm}$ is used for this task. In that case, $t_{rs}$ can be neglected. However, since it is intended to use a relative slow light source employing a power LED, $t_{rs}$ needs to be identified first. Power LEDs including driver are known to have typical bandwidths in the range from a few MHz to tens of MHz. Figure 3 shows the configuration for temporal measurements, comprising a signal generator Rigol DG5072 (Rigol Technologies Inc., Suzhou, China), a TC4452 driver (Microchip Technology Inc., Chandler, AZ, USA) including the LED as DUT, a Thorlabs PDA-10A 150 MHz transimpedance amplifier (TIA) PD module (Thorlabs Inc., Newton, NJ, USA), and an R&S HMO3004 digital oscilloscope (Rohde & Schwarz, Munich, Germany). The oscilloscope is directly providing the 10% to 90% rise time of the measured signal.

**Figure 4:**
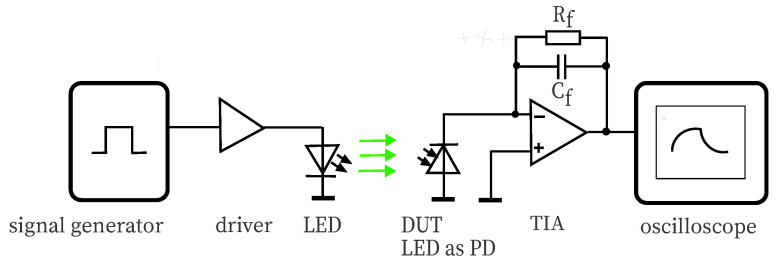
This block diagram depicts the configuration for determination of the rise/fall time of LEDs deployed as photodetectors. The source is configured as in Figure 3. The irradiated DUT is connected to a transimpedance amplifier. Its pulse shape is recorded by a digital oscilloscope.

For measuring the rise time of the DUT, the setup in Figure 4 uses the source introduced in Figure 3. The optical bandwidth of the sourcing LED should overlap well with the band of the DUT. The generated photocurrent is fed into a transimpedance amplifier THS4631 evaluation board (Texas Intruments, Dallas, TX, USA), which offers a gain bandwidth product (GBP) of 210 MHz and is configured with a feedback resistance $R_{f}$ of 47 kΩ and a feedback capacitance $C_{f}$ to accomplish a quality factor of approximately Q=0.7. The capacitance value *C* was measured with a Wavetek LCR55 m (Wavetek Corp., San Diego, CA, USA). Using a TIA calculator is very helpful at this point, available online at [23]. The evaluation of the response signal stored by the oscilloscope delivers the rise/fall time and provides an estimate of the achieved quality factor.

### 2.3. Spatial Measurements

**Figure 5:**
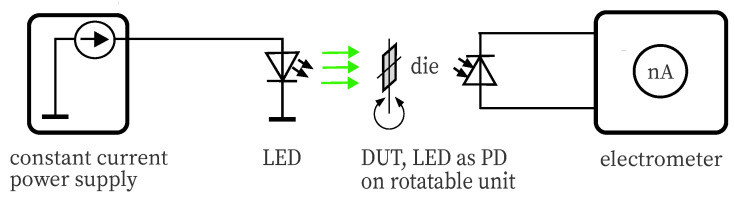
This illustration shows the principle system for determination of the directional characteristic of a photodetector. The DUT is rotated so that the irradiation hits the active area at a defined angle.

The angle of incidence (AOI) is an important parameter when dealing with optical systems. An optical bench for precise alignment and good repeatability is utilized. For spatial measurements, light from a stabilized LZ4 series LED source (Ledengin, San Jose, CA, USA) in blue, respectively, amber color with wavelength matching the DUTs, is used. The DUT is mounted on a rotatable device, to be able to adjust the angle of incidence, see Figure 5. Such setup is also known as a goniometer. The generated photocurrent is measured precisely with a Keithley 6517 electrometer in the nanoampere range. Measurements were conducted for a green and red Osram LED used as photodetector and for a green Lumileds Z LED to allow for minimal comparison within a series and between series, since no major deviations from the radiation characteristics as LED source are expected. AOIs between 0° and 80° taken in 10° steps were recorded. Based on the measurements with varying AOI, the FOV can be determined.

**Figure 6:**
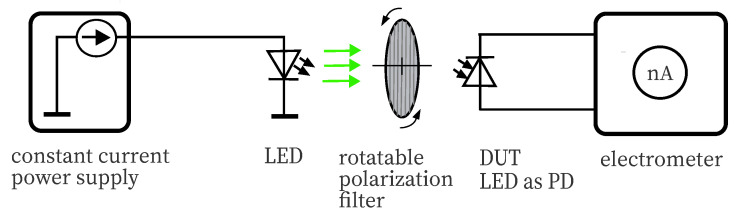
This graphic depicts the setup for checking a possible sensitivity to polarization. The light of a unpolarized LED source is passing a polarization filter. The polarization direction depends on the rotation position.

Another test is to check whether the DUT reacts unexpectedly to changes in the direction of polarization. For this purpose, non-polarized light from an LED source with the appropriate wavelength matching the DUTs passing band is filtered through a rotatable Edmund glass polarizer 53344 (Edmund Optics, Barrington, NJ, USA), see Figure 6. By turning the filter by 90°, the polarization direction can be changed from horizontal to vertical. Due to the effort, the full series was not examined, as DUTs red LEDs of both examined series were chosen exemplary. An amber-colored Ledengin LZ4 LED served as the source, operating in constant current mode under stable conditions. Photocurrent measurements were made directly with a Keithley 6514 electrometer, taken in 10° rotation increments.

## 3. Examination and Experimental Results

### 3.1. Analysis in the Spectral Domain

**Figure 7:**
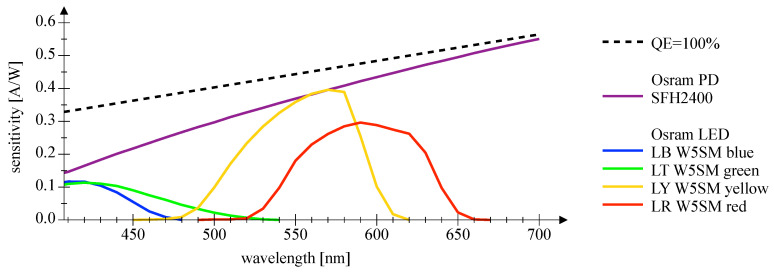
This plot shows spectral sensitivity measurements of Osram Golden Dragon series LEDs in four colors utilized as a photodetector. The measurements are compared with an Si PIN photodetector and its theoretical bound.

**Figure 8:**
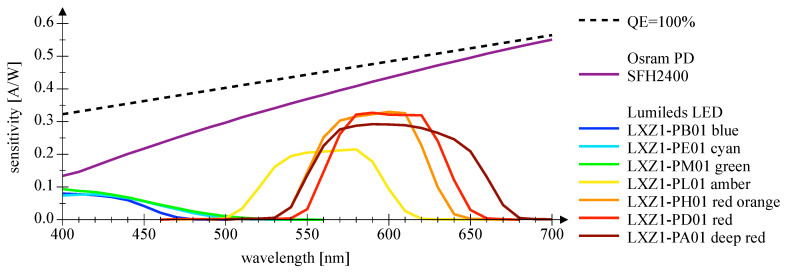
This plot shows spectral sensitivity measurements of Lumileds Z series LEDs in seven colors utilized as a photodetector. The measurements are compared with an Si PIN photodetector and its theoretical bound.

**Figure 9:**
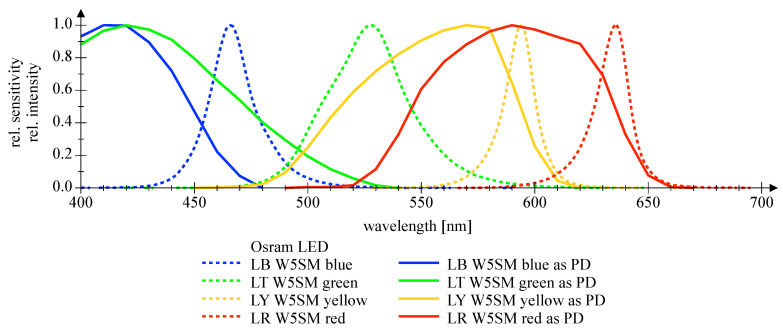
Normalized spectral measurements of Osram Golden Dragon series LED, deployed as emitter (dashed lines) and as detector (solid lines), respectively.

**Figure 10:**
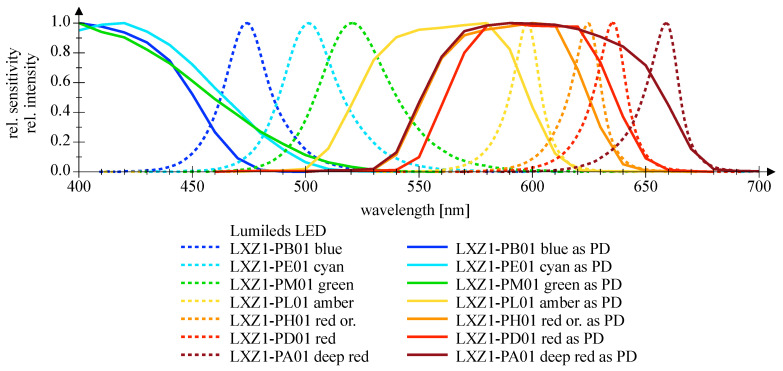
Normalized spectral measurements of Lumileds Z series LED, deployed as emitter (dashed lines) and as detector (solid lines), respectively.

**Figure 11:**
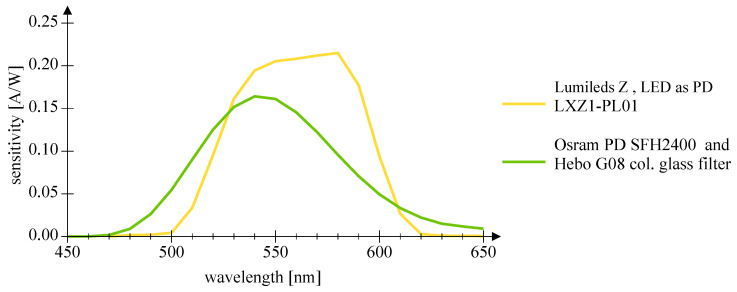
This diagram compares the spectral sensitivity of an LED deployed as PD with an Si PIN photodetector combined with a colored glass bandpass filter G08.

Figure 7 and Figure 8 depict the wavelength-dependent responsitivity of single-color power LEDs deployed as PDs. The sensitivity maximum of the DUTs is approximately 50% to 100% of the reference Si PIN PD at the appropriate peak wavelength, which is a comparatively high sensitivity. The theoretical responsitivity bound of an ideal Si PIN PD with a quantum efficiency (QE) of 100% is shown for reference purposes [24]. In Figure 9 and Figure 10, the spectral characteristics are normalized to compare the intensity as LED and the sensitivity as PD for two series of power LEDs in four, respectively, seven colors. The LED spectrum was measured at 300 mA forward current and 25 °C ambient temperature using a BTS 256 Spectrometer (Gigahertz Optik GmbH, Türkenfeld, Germany). Unfortunately, the full spectral bandwidth of blue to green LEDs as PDs can not be shown, as it is limited to wavelengths above 400 nm, due to the confined spectral range of the light source. Nevertheless, the position, the width, and the overlap of the spectral intensity respectively sensitivity can be identified. The spectral overlap of all DUTs is relatively small, which would result in a reduced efficiency if the same LED type would be used as transmitter and receiver. Visually, this effect appears to be more pronounced in the blue-green compared to the yellow-red color range, and the extent is difficult to estimate. Calculations for the yellow-red regime are giving spectral efficiencies of 56% to 63% in relation to imaginary overlapping peaks; for the blue-green range, only poor values of 7% to 18% can be assessed. Looking at pure bandpass filter features like width of the passing band and slope, LEDs deployed as PD can be an alternative to Si PIN PDs combined with a colored glass bandpass filter, see Figure 11 and [18]. Colored glass bandpass filters are only available on the market in the blue-green band, not in the yellow-red band. A thin film filter, however, generally delivers steeper slopes and can be designed over wide ranges with respect to the center wavelength and are much narrower in the passband, but with the disadvantage of a high price. Figure 11 compares the spectral sensitivity of an LED deployed as PD with an Si PIN photodetector combined with a colored glass bandpass filter G08 (Hebo Spezialglas, Aalen, Germany). Investigations of the influence of the temperature on the photodetectors responsivity have not been carried out, since they are known to be small and would not justify a considerable effort for a supposedly insignificant result. Information on the measurement uncertainties is given in Appendix A.

### 3.2. Analysis of the Temporal Domain

The approximate bandwidth (BW) resulting from rise/fall time measurements can be calculated by

(1) $$BW\approx \frac{0.35}{t_{r}}.$$

This equation holds for signals with approximately the same rise and fall time [25]. This condition is given for the LEDs under investigation. For an experimental determination of the rise time $t_{rd}$ of the detector, the rise time $t_{rs}$ of the source needs to be taken into account unless $t_{rs}\ll t_{rd}$. The measurable rise time $t_{rm}$ is the geometric addition of $t_{rs}$ and $t_{rd}$:

(2) $$t_{rm}=\sqrt{{t_{rs}}^{2}+{t_{rd}}^{2}}.$$

In the case of an LED used as source and a fast TIA-PD module as detector, with the smallest measured value $t_{rm}$ of 40 ns and a $t_{rd}$ of 2.3 ns for the used 150 MHz TIA-PD module, $t_{rd}$ is negligible and $t_{rm}$ approaches $t_{rs}$.

In Table 3, the measured values $t_{rm}$ of the LEDs used as emitter are shown, given the setup in Figure 3. The calculated bandwidth is in the expected range.

In Table 4, the measured values for $t_{rm}$ of the two LED series employed as PD are given; the corresponding setup is depicted in Figure 4. The source was matched to the spectral sensitivities, see Table 3 and Figure 7, Figure 8, Figure 9 and Figure 10. The rise time of the DUTs and the regarding bandwidth are calculated.

In Table 5, the measured junction capacitance of the LEDs and the bandwidth as result of the TIA simulation are tabulated. The simulated bandwidth based on capacitance measurement and the achieved bandwidth for LED employed as PD driving a TIA in a real LED sourced test setup are showing mostly good agreement. The deviation with respect to the determined bandwidths are caused by the difficulty to tune the TIA exactly to Q=0.7 due to the limited availability of small graded capacitors in the low picofarad range. Both series of LEDs employed as PDs are showing larger capacitances, resulting in longer rise times and lower bandwidths in the blue-green regime compared to the yellow-red regime. Information on the measurement uncertainties is given in Appendix A.

### 3.3. Analysis of the Spatial Domain

**Figure 12:**
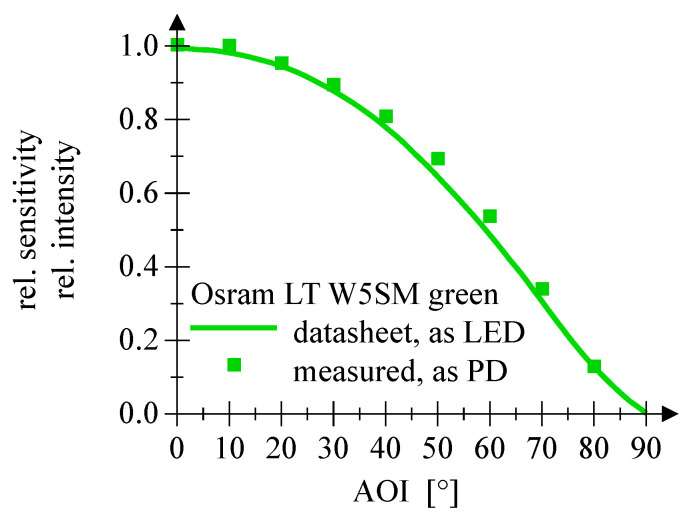
This plot shows the AOI comparing datasheet values and measured PD employment of a green Osram LED.

**Figure 13:**
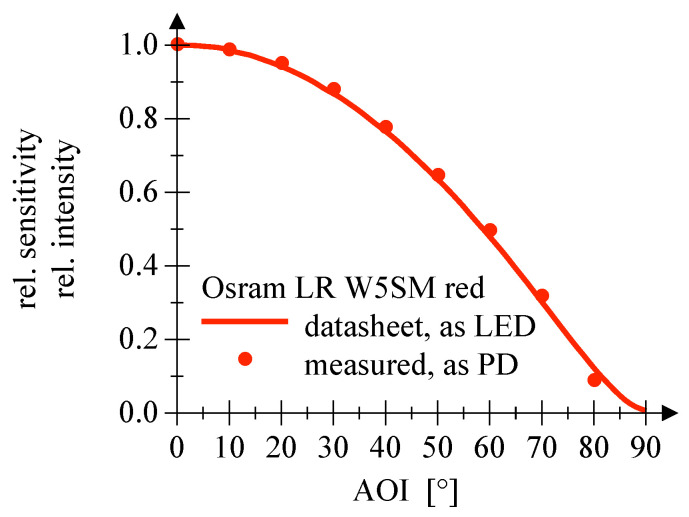
This plot shows the AOI comparing datasheet values and measured PD employment of a red Osram LED.

**Figure 14:**
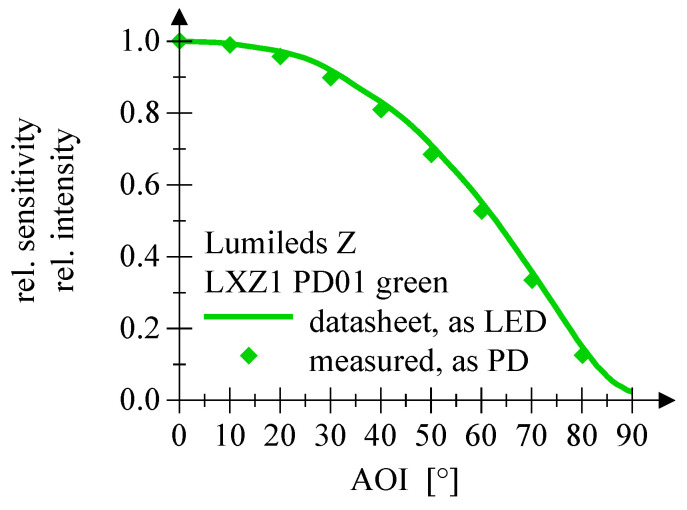
This plot shows the AOI comparing datasheet values and measured PD employment of a green Lumileds Z LED.

The measurement of the relative sensitivity as function of the angle of incidence for the LEDs employed as PD shows no significant deviation from the values given in the datasheet of the particular LED, cf. Figure 12, Figure 13 and Figure 14.

**Figure 15:**
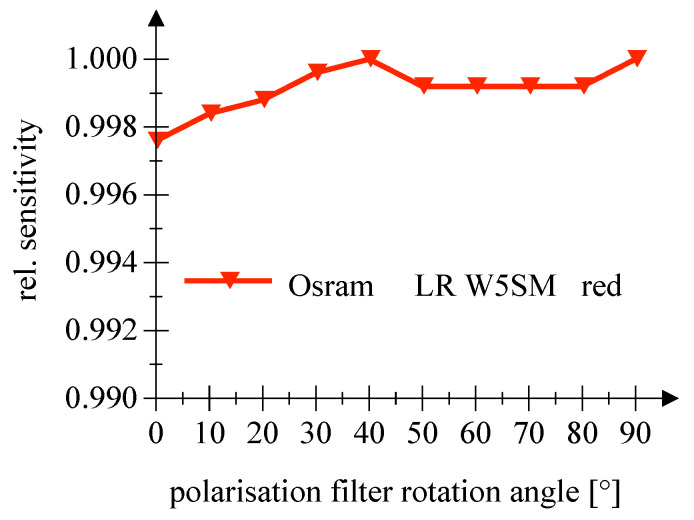
This figure shows the measured sensitivity as a function of the polarization angle for a red Osram LED employed as PD.

**Figure 16:**
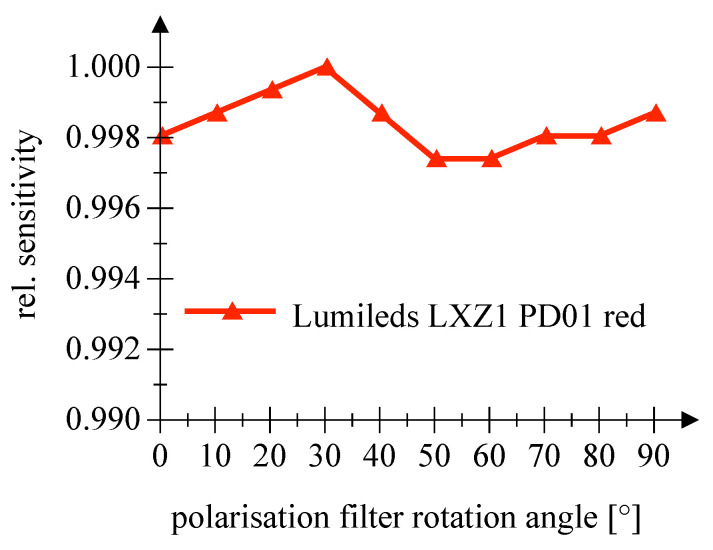
This figure shows the measured sensitivity as a function of the polarization angle for a red Lumileds LED employed as PD.

The examination of a possible influence of the polarization direction of the incident light on the sensitivity did not reveal any indications within the scope of the measuring accuracy, cf. Figure 15 and Figure 16. Please note the scaling. Information on the measurement uncertainties is given in Appendix A.

## 4. Conclusions

We experimentally investigated the spectral, temporal, and spatial characteristics of two single-color power LED series employed as PD. The examined LED series feature unexpected good responsitivity, not just compared to low-power LEDs studied in previous publications, but also compared to a common Si PIN photodetector and its theoretical sensitivity bound. The dual-use of the same LED as emitter and detector is possible, but will be accompanied by a relatively poor efficiency due to the small spectral overlap. This overlap is slightly better in the yellow-red regime compared to the blue-green regime. The well-known “green gap” of LEDs, centered around 550 nm, is still present for LEDs used as PDs, but shifted to lower wavelengths of approximately 500 nm, now presenting a “cyan gap” of sensitivity. One main characteristic of LEDs utilized as PD is to offer an inherent optical bandpass characteristic. This feature can be an alternative to the combination of PDs with secondary filters in thin film or colored glass technology. Particularly since colored glass bandpass filters are not available in the yellow-red region, off-the-shelf PD filter combinations are rare and thin film filters are expensive. The junction capacitance of the examined LEDs employed as PDs was found to be one to two orders of magnitude higher as compared to Si PIN PD reference; accordingly, the achievable bandwidth is reduced. The yellow-red regime offers a lower capacitance, thus allowing higher speeds as their counterparts in the blue-green area. The angle of incidence behavior of the LEDs under investigation was discovered to be the same when operated as emitter and detector, and no polarization direction dependence could be observed. LEDs used as PDs can serve as low-cost solutions in visible light communication, daylight filtered, or color-selective applications.

## Figures and Tables

**Table 1:** Key parameters of tested LEDs used as photodetectors.

Manufacturer	Type	Color	Wavelength, Dom. [nm]	Spec. Halfwidth, Typ. [nm]	Lum. Flux, Typ. [lm, @500 mA]
Osram	LB W5SM	blue	467	25	31
	LT W5SM	green	528	33	98
	LY W5SM	yellow	590	18	82
	LR W5SM	red	628	18	66
Lumileds	LXZ1 PB01	blue	470	20	38
	LXZ1 PE01	cyan	495	30	82
	LXZ1 PM01	green	530	30	118
	LXZ1 PL03	amber	599	20	56
	LXZ1 PH01	red orange	615	20	65
	LXZ1 PD01	red	632	20	52
	LXZ1 PA01	deep red	660	20	350 mW

**Table 2:** Key parameters of Si PIN photodetector used for comparison.

Manufacturer	Type	Blue Enhanced	Sensitivity, Range [nm]	Sensitivity, Max. [A/W, @850 nm]
Osram	SFH 2400	no	380...1100	0.65

**Table 3:** Measured values for 10% to 90% rise time $t_{rm}$ of investigated LEDs used as emitter.

Manufacturer	Type	Color	Wavelength, Dom. [nm]	$t_{\mathrm{rm}}$, Meas. [ns]	BW, Calc. [MHz]
Osram	LD W5SM	deep blue	455	40	8.75
	LT W5SM	green	528	63	5.56
	LY W5SM	yellow	590	130	2.69
	LR W5SM	red	628	70	5.00

**Table 4:** LEDs used as photodetector, measured rise time $t_{rm}$, calculated rise time $t_{rd}$ and calculated bandwidth BW, for TIA configured to approximately Q=0.7.

Manufacturer	Type	Color	$t_{\mathrm{rs}}$ [ns]	$t_{\mathrm{rm}}$ [ns]	$t_{\mathrm{rd}}$, Calc. [ns]	BW, Calc. [MHz]
Osram	LB W5SM	blue	40	490	488.3	0.72
	LT W5SM	green	40	470	468.3	0.75
	LY W5SM	yellow	63	139	123.9	2.83
	LR W5SM	red	70	182	168.0	2.08
Lumileds	LXZ1 PB01	blue	40	505	503.4	0.70
	LXZ1 PE01	cyan	40	410	408.0	0.86
	LXZ1 PM01	green	40	388	377.9	0.93
	LXZ1 PL03	amber	130	294	263.7	1.33
	LXZ1 PH01	red orange	70	177	162.6	2.15
	LXZ1 PD01	red	130	224	182.4	1.92
	LXZ1 PA01	deep red	130	274	241.2	1.45

**Table 5:** LEDs as photodetector, measured capacitance and simulated TIA BW for Q=0.7, GBP = 210 MHz and $R_{f}=47\;k\Omega$. For comparison: the reference Si PIN PD Osram SFH 2400 has a capacitance of 11 pF at zero reverse voltage, resulting in a simulated BW of 7.8 MHz.

Manufacturer	Type	Color	C, Meas. [pF]	$C_{f}$ [pF]	BW, Sim. [MHz]
Osram	LB W5SM	blue	950	5.5	0.86
	LT W5SM	green	680	4.7	1.02
	LY W5SM	yellow	105	1.8	2.58
	LR W5SM	red	235	2.8	1.73
Lumileds	LXZ1 PB01	blue	1600	7.2	0.67
	LXZ1 PE01	cyan	1120	6.0	0.80
	LXZ1 PM01	green	1270	6.4	0.77
	LXZ1 PL03	amber	420	3.7	1.30
	LXZ1 PH01	red orange	192	2.5	1.91
	LXZ1 PD01	red	226	2.7	1.76
	LXZ1 PA01	deep red	370	3.5	1.38

## Abbreviations

The following abbreviations are used in this manuscript:

AOI	Angle of Incidence
BW	Bandwidth
DUT	Device under Test
FOV	Field of View
FWHM	Full Width Half Mean
GBP	Gain Bandwidth Product
LED	Light Emitting Diode
PIN	Positive Intrinsic Negative
PD	Photodetector
QE	Quantum Efficiency
TIA	Transimpedance Amplifier
VLC	Visible Light Communication

## Appendix A

The main uncertainty of measurements in the spectral domain are the responsivity values given in the data sheet of the reference photodetector, produced by the uncertainty of irradiation measurements and variations in the series. Three photodetectors were measured and their mean was used for reference purposes. The influence of the accuracy of the photocurrent measurement, light source stability, mechanical positioning as well as the wavelength uncertainty of the monochromator and spectrometer are significantly lower. As the main source of uncertainty for the measurements in the temporal domain, the configuration of the feedback capacitor and the resulting quality factor can be identified. Timing and capacity values can be determined with relatively high accuracy. In the spatial domain, the measurements are relative photocurrent measurements, which can be performed with small uncertainties. The light source drift was controlled by alternating between zero position and the individual measuring position for each value taken. The manually rotatable positioning is subject to a certain error. Table A1 is giving an overview.

**Table A1:** Measurement uncertainties of most relevant parameters for the three domains examined.

Domain	Part	Measure	Unit	Uncertainty	Comment
spectral	electrometer	DUT photocurrent	nA	±0.2%	specs.
spectral	reference photodetector	responsivity	A/W	±20%	estimation
spectral	optical bench	DUT xyz-position	mm	±0.3	absolute
spectral	halogen light source	intensity drift	%/h	±1	estimation
spectral	monochromator	wavelength	nm	±0.5	specs., repeatability
spectral	spectrometer	wavelength	nm	±1	specs.
temporal	oscilloscope	rise time	ns	±0.5	specs, resolution
temporal	DUT	capacitance	pF	±0.2%	specs.
temporal	TIA	Q quality factor	-	±0.1	estimation
spatial	electrometer	DUT photocurrent	nA	±0.2%	specs.
spatial	LED light source	intensity drift	%/min	±0.1	estimation
spatial	polarisation filter	rotation	°	±3, ±1	absolute, repeat
